# Integrating Fat Graft with Blepharoplasty to Rejuvenate the Asian Periorbita

**DOI:** 10.1097/GOX.0000000000002365

**Published:** 2019-10-15

**Authors:** Juan C. Larsson, Tai-Yuan Chen, William W. Lao

**Affiliations:** From the * Department of Plastic and Reconstructive Surgery, Sanatorio Allende, Córdoba, Argentina; † Department of Orthopedic Surgery, Sanatorio Allende, Córdoba, Argentina; ‡ Department of Medical Education, Taipei Veteran General Hospital, Taipei, Taiwan; § Department of Plastic and Reconstructive Surgery, Chang Gung Memorial Hospital and Chang Gung University, School of Medicine, Taoyuan, Taiwan.

## Abstract

Contemporary periorbital rejuvenation is undergoing a paradigm shift to an approach that prioritizes volume preservation and/or augmentation. However, the technical difficulties of using traditional fat grafting techniques in this area and the distinct ethnic features make periorbital rejuvenation in Asians especially challenging. Here, the authors present their approach to enhance the periorbita and outcomes.

**Methods:**

A retrospective chart review was performed for 33 consecutive patients who underwent microautologous fat transplantation (MAFT) to the periorbita using the MAFT gun device combined with excisional blepharoplasty. Additionally, preoperative and postoperative photographs were analyzed by external evaluators to grade the aesthetic outcomes and fat resorption rates.

**Results:**

Three months after surgery, patients looked on average 5.4 ± 3.4 years younger, and the aesthetic result was graded 7.4 ± 2 in a one-to-ten scale. The fat resorption rate was 19.6% ± 3.5% at 3 months and 32.2% ± 3.9% after 12 months (range: 12–24 months; *P* = 0.007). The overall morbidity rate was 12% (4 patients), including 1 visible lump (3%), an overcorrection case (3%) in the lower eyelid, and 2 palpable lumps in the upper eyelid (6%) which were not visible. One case of lower eyelid hollowness required secondary fat grafting.

**Conclusions:**

Traditional blepharoplasty procedures can be combined with fat grafting techniques to address volume loss and tissue descend while keeping ethnic identity. A fat injection device like the MAFT gun is safe and effective and provides long-term predictable outcomes for fat grafting around the periorbital thin skin.

## Abstract 2

Supplemental Digital Content is available in the text.

## INTRODUCTION

In the Asian population, the most common tell-tale sign of aging is around the eyes.^1^ Asian eyelids have distinctive contours that differentiate them from white eyelids.^2^ The Asian upper eyelid has a very low eyelid crease and relatively low brow position, being their shape and contour typically very full.^3^ Additionally, Asian faces have weaker skeletal support, thicker skin, and heavier soft tissue, thus being more subjected to gravitational forces.^4^ These unique anatomical features determine the process of facial aging and dictate different goals in Asian rejuvenating lid surgery.^2,4^

Periorbital aging is a complex process involving tissue descent and deflation. Bony remodeling leads to a wider periorbital aperture which along with fat atrophy results in the appearance of protruding retroseptal fat pads, tear trough deformity, and negative vector.^5,6^ Traditional excision-based blepharoplasty procedures only remove excess soft tissues; they do not address the volume loss that often occurs in the upper eyelid sulcus and the tear trough area. Furthermore, they can lead to an exaggerated hollowed appearance, giving the impression of an “operated” look. Thus, to properly reverse all aging changes, the concept of “lift and fill” popularized by Rohrich et al^7^ and Pezeshk et al^8^ for facelift surgery should also be considered for the periorbital region. Treating for volume loss in the periorbita is just as important as addressing the excess skin and herniated fat.

With the current trends in plastic surgery, fat grafting has become the main autologous tool for facial volumization.^9^ Specifically by filling in the periorbital depressions, many surgeons have shown great restoration of the youthful transition between eyelid anatomical units with the brow and the cheek.^10–12^ But even in the most experienced hands, periorbital fat grafting carries a high risk of permanent adverse sequela.^13^ The unpredictable resorption rate and formation of permanent fat lumps can all lead to unfavorable results.^14,15^ To avoid these complications, Lin et al^10,16^ use a microautologous fat transplantation (MAFT) gun (Dermato Plastica Beauty Co, Kaohsiung, Taiwan), which allows smaller and more controlled fat parcel delivery.

The aim of this study is to describe our experience and outcomes in rejuvenating the periorbita in Asians through a combination of traditional excision blepharoplasty and volumetric supplementation of fat using the MAFT gun device.

## MATERIALS AND METHODS

A chart review was performed between January 2015 and January 2018 for 33 consecutive patients undergoing blepharoplasty with fat grafting to the periorbita by the senior author (W.W.L.) after approval from the institutional review board (No. 201800757B0). Patients excluded were those who received only excisional blepharoplasty procedures without fat grafting or presented with blepharoptosis, severe brow, or cheek ptosis, where ancillary procedures such as brow lift, facelift, or blepharoptosis corrections were performed. Those with a follow-up of <3 months were also excluded. All cases included in the study had abdomen as the single fat donor site (See Video 1 [online], which displays the fat harvest and preparation method and the MAFT gun loading technique.

**Video 1.** This video displays the fat harvest and preparation method and the MAFT gun loading technique.

## APPROACH TO ASIAN PERIORBITAL REJUVENATION

### Upper Eyelid

The upper eyelid was routinely evaluated for 3 factors: (1) volume deficiency or hollowness, (2) excess skin, and (3) protruding fat pads. Each aging change was considered an independent factor and was addressed in surgery. For patients with superior sulcus hollow, fat grafting was performed (See Video 2 [online], which displays the upper eyelid fat grafting method using the MAFT gun device.

**Video 2.** This video displays the upper eyelid fat grafting method using the MAFT gun device.

**Fig. 1.**
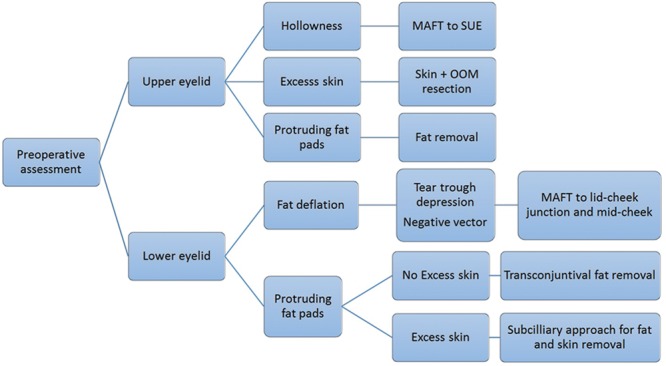
Comprehensive approach to upper and lower eyelid rejuvenation. OOM indicates orbicularis oculi muscle; SUE, sunken upper eyelid.

If excess upper lid skin was present, surgical excision of the skin and orbicularis oculi muscle was done. The amount and location of skin incision was determined by how much upper lid tarsal show the patient had and desired. For protruding fat pads, surgical trimming was done through the same incision (Fig. 1).

### Lower Eyelid–Cheek Complex

The lower eyelid was also assessed for the 3 aging factors: (1) the presence of tear trough depression or negative vector, (2) protruding retroseptal fat pads, and (3) excess skin. A flattened anterior cheek relative to the lower eyelid (negative vector) or a visible tear trough received fat grafting. The area for fat grafting was a semilunar or triangular area that extended from just above the tear trough to mid-anterior cheek (See Video 3 [online], which displays the lower eyelid fat grafting method using the MAFT gun device. If the orbital fat bulged anteriorly, beyond the surgeon’s perception of a smooth eyelid–cheek interface, retroseptal fat was removed. The presence of excess skin in the lower lid dictated the access route for fat removal. A subciliary incision was performed to resect skin and protruding fat pads, whereas a transconjunctival approach was used for patients with no skin excess (Fig. 1).

**Video 3.** This video displays the lower eyelid fat grafting method using the MAFT gun device.

### Outcome Evaluation

A retrospective photographic analysis and patient’s medical history review were conducted to evaluate patient outcomes. Chart reviews for fat grafting complications such as prolonged ecchymosis and swelling (>1 month), infection, overcorrection, and skin irregularities such as lumps or nodules were recorded. Undercorrection and need for additional fat grafting were also noted but not considered as complications.

**Fig. 2.**
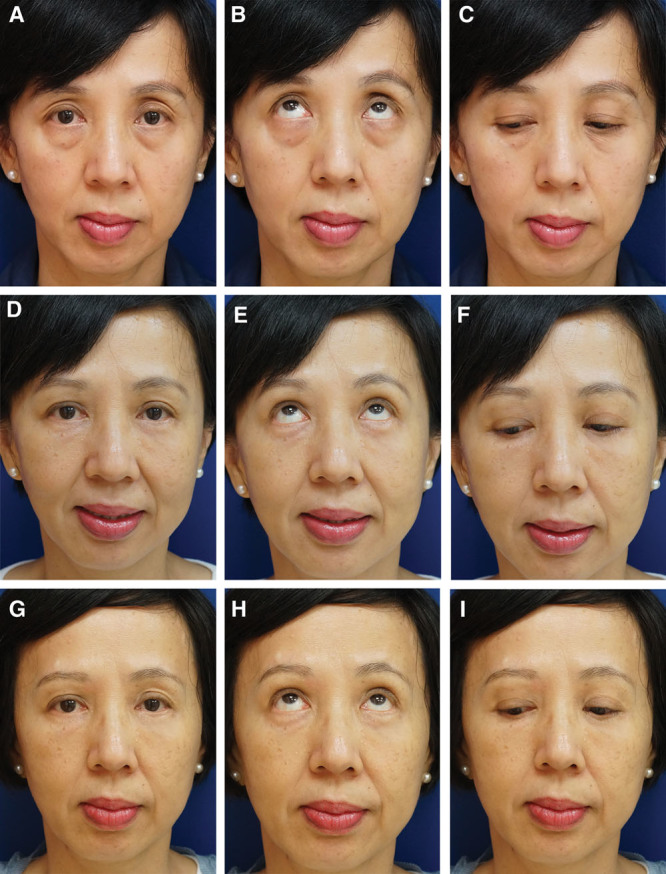
This 56-year-old woman presented for periorbital rejuvenation. Preoperative pictures in the frontal view are shown in primary (A), up (B), and down (C) gaze. The upper eyelid shows hollowing of the medial part with excess skin. The lower eyelid shows bulging fat pads, a sharp demarcation of the eyelid–cheek junction and moderate deflation of the malar region, especially in the anterior part. In the upper eyelid, skin resection and fat grafting of the medial orbit (2 ml on the right side and 2.5 ml on the left side) were performed. In the lower eyelid, 4 and 4.5 ml of fat were grafted on the right and left orbitomalar area, respectively. Additionally, pinch skin blepharoplasty and fat pad resection were performed to the lower eyelids. The 6-month postoperative pictures (D, E, F) show a fuller upper eyelid and a smooth lid–cheek transition which resulted in a shorter soft tissue vertical dimension of the orbit. In the 16-month postoperative pictures (G, H, I), the stability of the result is evident.

Seven plastic surgeons were invited to evaluate a series of paired photographs of each patient. All pictures were closed-up front view photographs of the face, matched to the best ability for size, proportion, background, and lighting (Fig. 2). The questionnaire included 3 questions and a grading scale to rate the results. Evaluators were asked to estimate the fat resorption rate from 0% to 100% in a visual analog scale after comparing pictures of 1 month after surgery versus 3 months after surgery and 1 month after versus the latest follow-up (including only patients with a minimum of 12-month follow-up). One month after surgery was chosen for comparison to exclude the effect of postsurgical swelling. Evaluators were blinded regarding the time of follow-up of each photograph. To assess the overall improvement, evaluators were asked to rate the results from 1 to 10 in a visual analog scale after comparing preoperative photographs versus 3-month postoperative photographs. The same photographs were used to answer the question: How many years younger does the patient look like?

Prism 7 (GraphPad Software, San Diego, Calif.) was used for statistical analysis. Aesthetic results were expressed by means ± standard deviation (SD) and resorption rates as ± standard error (SE). A *t* test was used to compare mean resorption rates. Statistical significance was defined as *P* < 0.05.

## RESULTS

The average age was 56 years old (range: 28–76 years); 27 patients (82%) were female and 6 (18%) were male. Twenty-six patients (78%) were operated under local anesthesia, 6 (18%) had intravenous sedation, and 1 (3%) had general anesthesia.

In our study population, 32 patients (97%) needed fat grafting to the lower eyelid, 14 (42%) to the upper eyelid, and 13 (39%) required both. The average amount of fat grafted to the upper eyelid was 1.6 ± 0.4 ml for the right side and 1.7 ± 0.5 ml for the left side. For the lower eyelid fat grafting, a mean of 3.3 ± 0.6 and 3.4 ± 0.6 ml was necessary to fill the right and left side, respectively (Table 1).

**Table 1.** Patient Characteristics, Operation Details, and Complications

Patient	Sex	Age N (Years)	Anesthesia	Procedure Combination		Fat Injection				Follow-Up N (Months)	Complications
				UE	LE	UERt, N (ml)	UELt, N (ml)	LERt, N (ml)	LELt, N (ml)		
1	F	59	Local	FG SR	—	1.5	1.5	—	—	4	Palpable lump (UE)
2	F	62	Local	SR FR	FG FR SR	—	—	3.5	3.5	6	
3	M	69	Local	SR	FG FR SR	—	—	4	4	24	
4	F	48	Local	SR	FG FR SR	—	—	2.5	2.5	24	
5	F	61	Local	—	FG FR SR	—	—	3	3	24	Visible lump (LE)
6	M	75	Local	SR FR	FG FR SR	—	—	3.5	3.5	22	
7	F	46	Local	—	FG FR	—	—	3.5	3.5	6	
8	F	64	IV	SR	FG SR	—	—	3	3	14	
9	M	61	Local	—	FG FR SR	—	—	3	3	14	Undercorrection (LE)*
10	F	55	Local	SR FR	FG SR	—	—	3.5	3.5	13	
11	F	52	Local	—	FG FR SR	—	—	3.5	3.5	11	
12	M	55	Local	—	FG FR SR	—	—	3	3	10	
13	F	34	Local	—	FG FR	—	—	3.5	3.5	10	Undercorrection (LE)
14	F	30	Local	—	FG FR	—	—	3	4	3	
15	M	53	Local	—	FG FR SR	—	—	4.5	4.5	8	Overcorrection (LE)
16	F	54	IV	SR FR	FG FR SR	—	—	4	4	3.5	
17	F	47	Local	—	FG FR SR	—	—	4	4	4	
18	F	58	IV	SR	FG FR SR	—	—	3	3	3	
19	F	47	IV	—	FG FR SR	—	—	4	4.3	3	
20	F	53	Local	FG FR SR	FG FR SR	2	2	4	4	3	
21	F	58	Local	FG SR	FG FR SR	2	2	3	3	12	
22	F	28	Local	FG SR	FG FR	1.5	1.5	3	3	18	Palpable lump (UE)
23	M	66	Local	FG FR SR	FG FR SR	2	2	3	3	18	
24	F	65	Local	FG FR SR	FG FR SR	2	2	3	3	17	
25	F	54	Local	FG FR SR	FG FR SR	1.5	2.5	2	2.5	15	
26	F	56	Local	FG SR	FG FR SR	2	2.5	4	4.5	16	
27	F	39	Local	FG	FG FR SR	1.5	1.5	3	3	10	
28	F	58	G	FG FR SR	FG FR SR	1.5	1.5	3	3	6	
29	F	65	Local	FG FR SR	FG SR	1	1	3	3	3	
30	F	59	Local	SR FR	FG SR	2	2	2	2	10	
31	F	76	Local	FG SR	FG FR SR	1	1	4	4	7	
32	F	68	IV	FG FR SR	FG FR SR	2	2	3.5	3.5	3	
33	F	65	IV	FG FR SR	FG FR SR	1	1	4.5	4.5	3	
Mean ± SD		56 ± 11.4				1.6 ± 0.4	1.7 ± 0.5	3.3 ± 0.6	3.4 ± 0.6	10.5 ± 6.9	

F, female; FR, fat removal; FG, fat grafting; G, general anesthesia; IV, intravenous sedation; LE, lower eyelid; Lt, left; M, male; Rt, right; SR, skin resection; UE, upper eyelid.

*Four milliliters of fat was injected to each LE in the revision procedure.

Among those patients who had fat grafting to the lower eyelid, the most common combination required was fat grafting together with skin resection and fat pad removal (75%). Other combinations included fat grafting with fat pad removal (12.5%) or with skin resection (12.5%). For those who had fat grafting to the upper eyelid, skin resection and fat pad removal were most commonly performed together (57.1%). A less common combination included fat grafting with skin resection (35.7%). Fat grafting alone was only performed in 1 case (7.2%; Table 1).

**Fig. 3.**
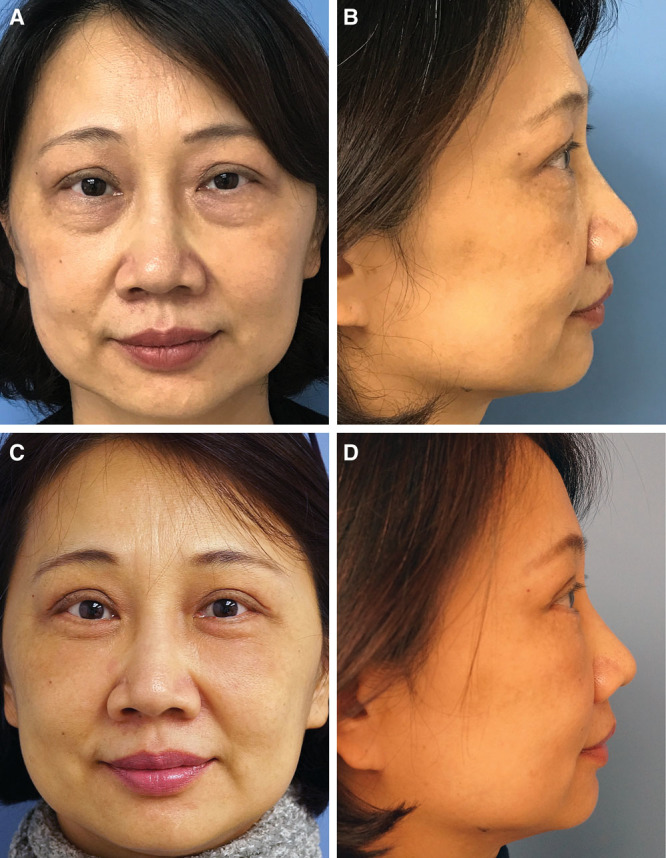
This 54-year-old woman presented for periorbital rejuvenation. Preoperative pictures in frontal view (A) show the protruding fat pads and excess skin present in the upper and lower eyelids. Note the moderately deflated malar region and demarcated orbitomalar groove. In addition, the preoperative picture in lateral view (B) shows a negative vector. Fat pad resection and skin blepharoplasty were performed in the upper and lower eyelids. A total of 4 ml of fat was grafted to the orbitomalar area on each side. Three-month postoperative pictures (C, D) show a blended lid–cheek junction and well-enhanced malar area after volume restoration. The height of the lower eyelid has been reduced giving an illusional “lift effect” of the cheek. The lateral view (D) shows a reversed negative vector, adequate anteroposterior malar projection, and a restored convex profile of the lower periorbital complex.

The overall morbidity rate was 12% (4 patients) after an average follow-up of 10.5 ± 6.9 months (range: 3–24 months). Among those who received fat grafting to the upper eyelid, 2 patients (14%) presented with palpable but not visible lumps in one eyelid each. Two patients (6.2%) who received fat grafting to the lower eyelid showed slight contour deformities in one eyelid each including one visible lump and one slight overcorrection (Fig. 3). Additionally, 2 cases (6.2%) of lower eyelid augmentation complained about undercorrection. Of these, 1 patient requested a secondary procedure where 4 ml of fat was grafted to each lower eyelid with successful results. No case of prolonged ecchymosis and swelling or infection was encountered. Most patients could return to social activities by the end of the second week and all of them by the third week.

At 3-month follow-up, patients looked 5.4 ± 3.4 years younger and their aesthetic result was graded 7.4 ± 2 in a one-to-ten scale. When 1-month follow-up photographs were compared with 3-month photographs the fat resorption rate was 19.6% ± 3.5%. On follow-up longer than 12 months, the fat resorption rate rose to 32.2% ± 3.9% (*P* = 0.007). The mean follow-up for the latter group was 17.7 ± 4.1 months (range: 12–24 months).

## DISCUSSION

**Fig. 4.**
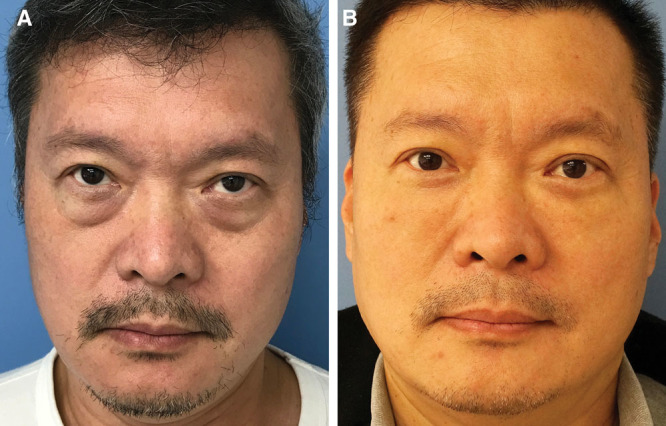
This 59-year-old man presented for periorbital rejuvenation. The preoperative pictures (A) show protruding fat pads in the lower eyelid, a demarcated lower orbital rim, and deflated anterior cheek. The bulging fat pads were removed, and a strip of skin from the lower eyelids and 4.5 ml of fat were grafted to each side. The 15-month postoperative picture (B) shows a blended lid–cheek junction and enhanced lower eyelid appearance. However, a subtle and homogenous bulging is evident at the right lid–cheek junction.

Periorbital rejuvenation is a key element in facial rejuvenation. By treating only the periorbita, the overall facial appearance can improve substantially (Figs. 2, 4). The aging process of the periorbita, however, has individual variations. Soft tissue excess and volume depletion could both be present albeit in different severity. Hence, tailoring the surgical approach is critical to address each component of the aged periorbita, either by using traditional blepharoplasty techniques, fat grafting for augmentation, or a combination of both.

Our Pubmed review of the English literature shows increasing evidence that supports the benefits of fat grafting in periorbital rejuvenation, either alone or in combination with traditional blepharoplasty procedures (Table 2). There are few reports on the Asian population.^10,17^ Most authors agree that these procedures can be accomplished safely and comfortably under local anesthesia with or without oral or intravenous sedation,^14,16,18–24^ though a few prefer general anesthesia.^11,12,21^ However, the choice of the best fat grafting method remains controversial. In general, most surgeons prefer using manual pressure for fat harvest^13,22–26^ and to take the fat from the abdomen^10,13,17–23,26,27^ or upper medial thigh.^11–13,19,21,27^ Less common donor sites such as the inner knee,^21,24^ hips,^26^ or other diet resistant areas^25^ have also been reported. Regarding fat preparation, the preferred method is centrifugation,^10–12,14,18–20,24–26^ though filtration,^13,22^ washing,^23,27^ or filtration with washing^21^ has been proposed. Few articles report on complications, outcome ratings, and patient satisfaction. Specifically, no data based on objective measures were found in the current literature on resorption rate after periorbital fat grafting.

**Table 2.** Literature Review: Periorbital Fat Grafting

Author/Year of Publication	Level of Evidence	Ethnicity	Patients	Anesthesia	Fat Harvest		Fat Preparation	Fat Injection	Follow-Up	
	(1–5)		N	Type/Solution	Donor Site	Harvest Method/Syringe/Cannulae	Processing Method	Cannula Size and Type	Months	Outcomes
Trepsat^24^/2003	5	Whites	500	Local/sedation	Knee, abdomen, buttocks, and back	MSP 1.5 ml/10 ml/1 mm	Centrifugation 3,000 rpm/3 min	19G	NS	Lumps 1%, infection 0.2%
Kranendonk and Obagi^26^/2007	5	Whites	250	Local	Abdomen, hips	NS	Centrifugation 3,000 rpm/2 min	Coleman* N°2	NS	Lumps 1.6%, infection 0.4%
Holck and Lopez^18^/2008	5	Whites	NS	Local/sedation	Abdomen, thigh	MSP/NS/NS	Centrifugation 3,000 rpm/30 s	Coleman* N°1 and N°2	NS	NS
Ciuci and Obagi^14^/2008	5	Whites	NS	Local/sedation	Diet resistant area; MAFT	MSP 1–2 ml/NS/NS	Centrifugation 1,286*g*/2 min	Coleman* N°2	NS	NS
Buckingham et al^19^/2010	5	Whites	NS	Local/sedation	Abdomen, thigh	MSP/30 ml/3 mm (Tulip†)	Centrifugation 3,000 rpm/2–3 min	0.9–1.2 mm	NS	NS
Park et al^17^/2011	4	Asians	41	NS	Thigh, abdomen	**NS**	NS	NS	4.7 (8–18)	Lump 4%, undercorrection 4%, fat resorption 20%–30%
Serra-Renom and Serra-Mestre^20^/2011	4	Whites	142	Local/sedation	Abdomen	MSP /NS/1.6 mm	Centrifugation 3,000 rpm/3 min	17G	24	Undercorrection 9.1%, asymmetry 0.7%, no infection, satisfaction: 3.91/4
Tonnard et al^21^/2013	4	Whites	500	General/local	Abdomen, knee, thigh	MSP/NS/2 or 3 mm	Filtration and washing (through a nylon cloth with 0.5 mm perforations, rinse with saline)	0.7 - 0.9 mm	16 (3–39)	Prolonged swelling 7% >1 month, scleral show 1%, no infection, overfilling or asymmetries
Collar et al^27^/2013	5	Whites	NS	Local / sedation	abdomen, thigh	Triport Harvester/10 ml/NS	Washing (with lactated Ringer's solution through strainer)	0.7 or 0.9 mm (Tulip†)	NS	NS
Massry and Azizzadeh^13^/2013	5	Whites	NS	Local	Abdomen, medial and lateral thigh	MSP2cc/10 ml/2.1 mm (Tulip†)	Filtration (Telfa dressing for 10 min)	0.9 mm (Tulip†)	NS	NS
Marten and Elyassnia^25^/2015	5	Whites	NS	Local/sedation	Areas resistant to diet and exercise	MSP 2.1/10 ml/2.4 mm (Tulip†)	Centrifugation 1,000 rpm/1–3 min	22G	NS	NS
Lin et al^10^/2016	4	Asians	34	Local/sedation	Abdomen	NS/NS/2.5 mm	Centrifugation 1,200 rpm/3 min	MAFT§ gun 18G (1/240 ml per parcel)	18.5	Undercorrection/touch up 12%
Ramil^22^/2017	4	Whites	32	Local/sedation	Abdomen	MSP/30–50 ml/NS	Filtration	0.9 mm	11	Prolonged edema 3.6%, no lumps, satisfied 97%
Pezeshk et al^12^/2017	5	Whites	NS	General	Thigh	NS	Centrifugation 1,200 rpm/1 minute. Emulsification (Tulip) 50 times pass.	1 mm	NS	NS
Rohrich et al^11^/2018	5	Whites	NS	General	Thigh	NS	Centrifugation 1,200 rpm/1 min. Emulsification (Tulip) 50 times pass.	0.9 mm (Micrins‡)	NS	NS
Demetriades et al^23^/2018	5	Whites	NS	Local/sedation	Abdomen	MSP/30 ml/2.1 mm (Tulip†)	Washing (Puregraft System)	18G (Tulip†)	NS	NS
Lao and Larsson^1^/2018	4	Asians	33	Local/sedation	Abdomen	MSP 25 ml syringe	Filtration (Telfa Dressing)	MAFT§ gun 18G (1/60 ml per parcel)	10 (3–24)	Visible lump 3%, palpable lump 6%, overcorrection 3%, undercorrection 6%, fat resorption 19%–32% (at 3 months and 1 y), satisfied 97% with one time fat grafting, 3% required touch-up

G, gauge; MSP, manual suction pressure with syringe; NS, not specified; rpm, revolutions per minute.

*Coleman, Byron Medical, Tucson, Ariz.

†Tulip Medical Inc, San Diego, Calif.

‡Micrins Medical Inc, Lake Forest, Ill.

§MAFT, microautologous fat transplantation gun (Dermato Plastica Beauty Co, Kaohsiung, Taiwan).

Puregraft, Solana Beach, Calif.

Telfa, Kendal Healthcare Products Company, Mansfield, Mass.

Fat injection to the periorbital area is a demanding procedure with a low margin for error. It is most commonly done by exerting manual pressure on a 1 ml syringe attached to 0.7 to 1.2 mm microcannulae (Table 2). Some technical aspects should be emphasized to add more safety to the procedure. First, the injection plane should remain deep to the orbicularis oculi muscle. Injecting in the preperiosteal plane to fill the deep fat compartments further adds more safety to the procedure.^11–13,17,19,21–25,27^ The deeper the fat injections are, the less chance of skin irregularities. This is especially critical in the periorbita as the skin is thin and has little overlying tissue. Even in Asians, where the skin is thicker compared with Westerners, postinjection lumps can appear despite our best effort (Table 1). Manual massage immediately after injection helps to ensure a smoother grafted surface. Second, to prevent “sausaging,” we prefer a criss-crossing technique by injecting fat from 2 different entry points keeping the cannula as perpendicular as possible to the long axis of the targeted area (See Video 3 [online], which displays the lower eyelid fat grafting method using the MAFT gun device. Third, the cannula passage should be gentle, and the tip should be palpated or visualized at all times. Placing the nondominant index finger at the level of the orbital rim limits the cannula passage preventing damage to the eye globe. Finally, we prefer to use fat delivery devices such as the MAFT gun to precisely control the size and location of each fat droplet. In the current study, volume depletion in the periorbita was addressed accurately and safely by using the MAFT gun, as evidenced by our favorable results. The few cases of irregularities found in our series appeared medially in the orbit and could be attributed to superficial fat injection during our early experience. From our experience, the medial periorbita, which encompasses the tear through, and the medial upper eyelid sulcus are more susceptible to unsightly contour problems. Conversely, the thicker skin and subcutaneous tissue of the lateral sub-orbicularis oculi fat (SO OF) and lateral upper eyelid sulcus in Asians relative to its medial counterparts makes the lateral periorbita more forgiving in terms of contour irregularities.

**Fig. 5.**
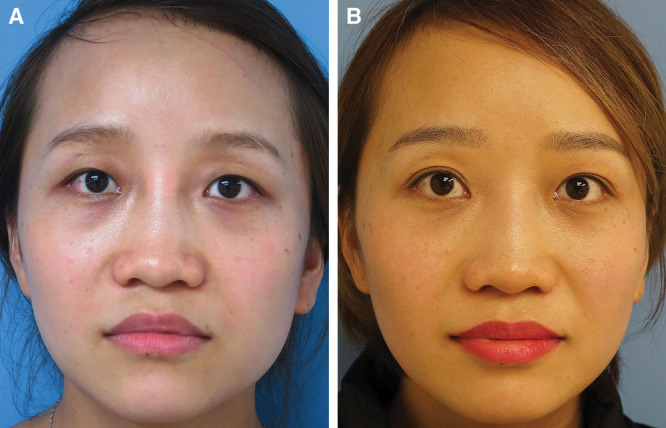
This 28-year-old woman presented for periorbital enhancement. The preoperative picture shows hollowness around the eyes conveying a sad and older appearance (A). Conservative transconjunctival fat pad resection and fat grafting (1.5 ml to each upper eyelid and 3 ml to each lower eyelid) were performed. The 18-month postoperative picture (B) shows a stable long-term enhancement of the periorbita.

In the present study, the fat resorption rate at 3 months compared with 1 month was 19.6% ± 3.5%. We choose to examine photographs at 3 months because clinically this is the time when we find that fat resorption tends to stabilize. However with our data, we learned that fat continues to have visible resorption beyond 3 months but just at a much slower rate (32.2% ± 3.9% on follow-ups longer than 12 months; Figs. 2, 4, 5). One should consider that these data are based on photograph comparison by plastic surgeon observers who were blinded about the time of follow-up. A correlation between preoperative imaging, total amount of grafted fat, and sequential imaging postoperatively could provide a more objective quantification of fat resorption. Nonetheless, our results are comparable with the qualitative observation of 20% to 30% resorption rate reported by Park et al,^17^ who recommended an overcorrection of the same magnitude anticipating for this long-term loss. However, considering the variability of fat resorption among patients and the rare necessity of revision augmentation procedures in our series, we believe overcorrection should be avoided when using our blepharoplasty combined approach. All patients are counseled on the likelihood of a second fat transfer procedure, although this rarely happens. Therefore, we suggest the endpoint of MAFT to be the disappearance of the upper sulcus hollow and a smooth transition of the lid–cheek junction. In our population, an average of 1.6 to 1.7 ml and 3.3 to 3.4 ml of fat injection were necessary to recontour the upper and lower eyelids, respectively.

Asian anatomy poses a different challenge when restoring volume on the upper eyelids. Westerners have a more prominent supraorbital arch, and the distance between the eyebrow and the upper eyelid margin is usually quite close.^25^ Their sunken upper eyelid is more tolerated due to the deeper upper sulcus, more superior eyelid crease, and thinner eyelid soft tissue at base compared with Asians.^25^ In Asians, the projections of the supraorbital arch and eye are similar, and the distance between the eyebrow and the eyelid margin is bigger. Volume loss usually appears as a limited dent over the already convex surface of the bulging eyelid instead of the hollow patterns above the tarsus seen in Westerners.^22^ This depression creates an apparent longer eyelid–brow distance, accentuating the aging eyelid. By fat grafting this area, the deep-set skin is brought up, restoring the natural fullness and smooth convexity of the upper eyelid and blending the eyelid–brow transition zone.^28^ Moreover, it results in a shortened eyebrow–eyelid distance, which gives the patient a more youthful appearance, while respecting and further highlighting her ethnic features (Figs. 2, 5).^10^

To fat graft the lid–cheek junction, we prefer to tailor the amount and location of the fat graft based on the deflated areas demarcated on examination as advocated by Marten and Elyassnia,^25^ rather than targeting any specific fat compartment as proposed by others (See Video 3 [online], which displays the lower eyelid fat grafting method using the MAFT gun device.^9,29^

Interestingly, a three-dimensional photographic analysis by Schreiber et al^30^ showed that the surface change after mid-cheek compartmental fat grafting resembled the shape of a boomerang, which matches the semilunar-shaped depleted area demarcated preoperatively at the lid–cheek interface in our patients. As demonstrated in our study, fat grafting this target area is safe and effective to soften the bony infraorbital contour, blend the lid–cheek transition zone, and project the malar prominence (Fig. 3). This corrects the “V deformity” and negative vector, reduces the height of the lower eyelid, and gives an illusional “lift effect” of the cheek (Fig. 2). This observation further supports speculation by Lambros^31^ and Pessa et al^32^ that in some patients, relative anteroposterior shifts in volume play a more dominant role in mid-facial aging than soft tissue descent. From our observations, it seems that most of our patients had an overall improvement of the malar region just by fat grafting the lid–cheek junction without the need for more extensive malar fat grafting (Fig. 2–5). An additional advantage of fat grafting to the lid–cheek junction during lower blepharoplasty is that it recruits eyelid skin and provides additional support to the lower eyelid. This reduces the risk of ectropion making this approach safer compared with skin resection alone. Conservative skin resection reduces fine wrinkles and further augments the fat grafting filling effect by tightening the eyelid skin. Although Lin et al^10^ showed good results by fat grafting smaller droplets of fat to the pretarsal and preseptal area, this might result in visible or palpable lumps because there is practically no fat between the orbicularis oculi muscle and the overlying eyelid skin.

To avoid unnatural results after upper blepharoplasty, especially in Asian patients, the upper eyelid crease should be kept between 5 and 7 mm from the ciliary margin both in men and in women.^33^ In Asians with a defined upper eyelid crease, resecting the orbicularis oculi muscle would be more advantageous to reproduce the tarsal fixation to the skin and levator aponeurosis.^28^ For patients with a well-positioned brow, it is better to avoid brow lifts to help preserve the proportional height of the crease which is a characteristic Asian feature. Patients with severe degrees of brow ptosis may need ancillary lifting procedures. In elderly patients, blepharoptosis is commonly encountered. These patients frequently present with upper eyelid pseudo-hollow caused by brow elevation. Frequently, just by correcting blepharoptosis, the upper eyelid hollow is resolved with relaxation of the brow, precluding the need for fat grafting.

## CONCLUSIONS

Periorbital aging is often a multifactorial process involving both volume loss and tissue descent. Combining fat grafting with traditional blepharoplasty techniques can address both aging changes while keeping ethnic identity. In the Asian population studied, the need for fat grafting becomes most evident starting the fifth decade of age. A fat injection device like the MAFT gun is effective and provides long-term predictable outcomes, but it is not without potential complications for fat grafting around the thin skin of the periorbita.
